# Usage of Children’s Makeup and Body Products in the United States and Implications for Childhood Environmental Exposures

**DOI:** 10.3390/ijerph20032114

**Published:** 2023-01-24

**Authors:** Eleanor A. Medley, Kendall E. Kruchten, Miranda J. Spratlen, Maricela Ureño, Anabel Cole, Rashmi Joglekar, Julie B. Herbstman

**Affiliations:** 1 Columbia Center for Children’s Environmental Health, Department of Environmental Health Sciences, Columbia University Mailman School of Public Health, New York, NY 10032, USA; 2 Earthjustice, Toxic Exposure and Health Program, Washington, DC 20001, USA

**Keywords:** cosmetics, environmental justice, exposure science, children

## Abstract

There is growing evidence of toxicity associated with ingredients found in cosmetics and personal care products. Children’s makeup and body products (CMBPs) are widely marketed to children throughout the US; however, little is known about how and why children use them. We administered a survey to parents/guardians of children aged ≤12 years about the use of CMBPs. Among all the children (*n* = 312) of survey respondents (*n* = 207), 219 (70%) have used CMBPs in their lifetime. Older children used CMBPs at higher rates than younger children, and female children used CMBPs at higher rates than male children. Children of Hispanic/Latinx parents/guardians used CMBPs more often and for shorter durations and a greater proportion used lip, hair, and fragrance products than children of non-Hispanic parents/guardians. Approximately half the children that use CMBPs were reported to use them with play intentions. Compared to children of non-Hispanic parents/guardians, children of Hispanic/Latinx parents/guardians reported more play motivations for CMBP use. Using qualitative analysis approaches, responses suggest CMBPs are commonly used for fun or play activities. This mixed methods analysis serves as an introduction to understanding early life exposures to this unique and understudied class of products.

## 1. Introduction

While cosmetics are typically associated with adult use, similar products are also widely marketed to children. In addition to more traditional makeup such as eyeshadow and lipstick, children may also use body products such as face paint, body glitter, nail polish, hair gel, and perfume or cologne. These children’s makeup and body products (CMBPs) incorporate features such as bright colors, animals, and cartoon characters to attract the attention of children. Social media platforms that children may use are also being increasingly utilized to advertise these products [1].

CMBPs are currently regulated at the federal level as cosmetics by the U.S. Federal Drug Administration (FDA) under the 1938 Federal Food, Drug, and Cosmetics Act (FFDCA) and the 1967 Fair Packaging and Labeling Act (FPLA) [2,3]. However, current safety regulations have been widely criticized as inadequate [4]. Major gaps in the regulation include a lack of mandatory pre-market safety approvals for products and ingredients other than color additives, lack of transparency in fragrance ingredients, limited recall of products, and under-reporting of adverse events [5,6]. Additionally, cosmetics are often marketed with vague terms such as “natural”, “organic”, “non-toxic”, and “hypoallergenic”, which are not defined by the FDA for cosmetic products [7,8].

In part due to these regulatory limitations, ingredients of concern have been found in adult cosmetics and CMBPs. For example, heavy metals such as lead, cadmium, and chromium have been found in children’s face paints [9]. Endocrine disruptors such as phthalates, parabens, and perfluorochemicals (PFCs) are also widely used in adult cosmetics and CMBPs [10,11,12]. Even when concentrations of individual chemicals are low in products, the potential for interactive effects from multiple toxicants is important to take into consideration [13]. Allergic reactions, such as contact dermatitis, are some of the most frequently cited negative health outcomes associated with the use of cosmetics [14]. Natural rubber, fragrances, preservatives, dyes, and metals are classes of allergens commonly found in cosmetics [15]. Rastogi et al. (1999) found that in certain CMBPs, fragrance allergens were frequently detected, occasionally at unsafe concentrations [16].

Children are particularly vulnerable to adverse health risks associated with makeup and body products. Behavioral patterns such as hand-to-mouth activity may increase exposure to products through ingestion. Additionally, children’s small body size, rapid growth rate, developing tissues and organs, and immature immune systems make them biologically susceptible to the effects of toxicants [17]. Childhood exposure to harmful makeup and body product ingredients can also be considered an environmental justice issue, as communities of color may be more likely to use these products [18,19,20]. For example, Li et al. (2002) reported that African American parents and their children used hormone-containing hair products at higher rates than participants of all other races/ethnicities [21]. McDonald et al. (2018) found that the use of some hair products at a young age is associated with earlier puberty, a risk factor for breast cancer [22]. Sprinkle (1995) found that the use of eye makeup imported from Pakistan was associated with higher blood lead levels in Pakistani and Indian children in California [23]. Marginalized communities experiencing disparate levels of exposure, compounded by sensitive periods of exposure, represent an environmental injustice that warrants further investigation.

Despite reasons for concern about CMBPs, little is known about how such products are used. This study aims to learn more about how CMBPs are used by children 12 years of age and younger in the United States. This study investigated sociodemographic characteristics, product exposure, product usage behaviors and motivations, and product purchasing behaviors through a parent/guardian questionnaire. While adults use makeup and body products for beautification, children may use them for different reasons but still experience resulting body burdens of environmental toxicants. We hypothesized that children aged ≤12 years use CMBPs primarily for play as opposed to beautification purposes. With a better understanding of how CMBPs are used, regulators may be able to improve product safety standards and reduce exposure in these vulnerable populations.

## 2. Methods

### 2.1. Survey Administration

We created a 39-question survey about the use of CMBPs using Qualtrics (version June–July 2021), with the goal of developing a survey that would take under 10–15 min to complete [24]. Parents/guardians aged ≥18 years with at least one child aged ≤12 years that live in the United States were eligible to complete the survey. Study participants were recruited from August 2021 to April 2022 through purposive sampling using email, social media (parental and neighborhood Facebook groups), and flyers placed at local businesses, schools, clinics, and community facilities in New York City, the Greater Boston Area, and the Greater Houston Area. Face-to-face recruitment was also conducted at events and locations in New York City. Participants were encouraged to share the survey with other eligible parents/guardians they knew. Study participants independently completed the survey on their personal devices. The survey was offered in English and Spanish. The study was approved by the Institutional Review Board at the Columbia University Irving Medical Center.

### 2.2. Survey Content

#### 2.2.1. Sociodemographic Characteristics

Sociodemographic characteristics of survey participants and their children were self-reported through survey questions about the state of residence, type of resident community (rural, urban, suburban), parent/guardian age, parent/guardian gender, parent/guardian race/ethnicity, parent/guardian education, household income in 2019 (pre-COVID-19 pandemic), number of people in the household, child age, child gender, and child race/ethnicity. 

#### 2.2.2. Definition of Children’s Makeup and Body Products

The survey informed participants that CMBPs are intended to be used primarily by children 12 years of age and younger. The survey also provided the following categories of children’s makeup and body products with examples:

- Body: face paint, body paint, temporary tattoos, stencils, body glitter, stick-on jewelry, tanning lotion, bath bombs.
- Eye: eye shadow, eye liner, mascara, eyebrow pencil, false eyelashes.
- Lip: lip gloss, lip stick, lip tint, lip liner.
- Face: foundation, concealer, powder, blush, bronzer, primer, contour, highlight, face masks.
- Nail: nail polish, nail stickers, fake nails, press-on nails.
- Hair: hair sprays, spray-on hair color, hair gel, hair styling mousse/creams, hair glitter.
- Fragrances: perfume, cologne, body spray.

We did not include products with primarily cleansing or medicinal purposes nor hair relaxers or skin-lightening products, in the definition of CMBPs.

#### 2.2.3. Child Exposure

Four questions addressed child exposure to ingredients and potential contaminants in CMBPs. These questions asked if the child has ingested CMBPs in the last year, how often parents read the ingredients list before purchasing CMBPs, how frequently the child used CMBPs in the last year, and for how long children typically wore CMBPs when they used them in the last year.

#### 2.2.4. Product Usage Behaviors and Motivations

Eight questions addressed children’s product usage behaviors and motivations. These questions asked if the child has ever used CMBPs, what type of CMBPs they use (according to the body part they are made for—see definitions above), what proportion of total CMBPs used by the child in the last year would be considered children’s products rather than adult products, who applies the CMBPs, how often they wore CMBPS outside of the home in the last year, and in what settings the child uses CMBPs. Parents were also asked to “rate on a scale of 1–10, how much the child uses CMBP for play versus for beautification in the last year (1 indicating all play and 10 indicating all beautification)” and had the option to describe in their own words how their children 12 and under use CMBPs.

#### 2.2.5. Product Purchasing Behaviors

Seven questions addressed product purchasing behaviors. These questions asked how the child was introduced to CMBPs, how much packaging influences the child’s interest in CMBPs, what the packaging of the CMBPs looks like, how the CMBPs are obtained, at what type of retailer the CMBPs are purchased, and how frequently the CMBPs are purchased.

### 2.3. Data Analysis

#### 2.3.1. Survey Responses

Survey responses were excluded if they were marked as “spam” by Qualtrics which occurs if multiple identical responses are received from the same IP address within a 12 h period (*n* = 172). Other survey responses were also excluded if they were one of five or more responses with identical demographics and had start times within 15 min of each other (*n* = 255). Incomplete responses were excluded from the analysis (*n* = 70). Responses were also excluded from analysis if participants reported having 0 children aged ≤12 years (*n* = 9). We used 207 total responses for analysis. The geographic distribution of survey responses was visualized with QGIS (version 3.10.14 with GRASS 7.8.5) [25]. 

#### 2.3.2. Quantitative Analysis of Multiple-Choice Questions

Descriptive statistics and hypothesis tests were run with R in RStudio (version 1.4.1725) [26]. Product use behaviors and motivations were compared across sociodemographic groups using Wilcoxon and Kruskal–Wallis tests for numeric variables, Chi-square tests for categorical variables with sufficient cell sizes, and Fisher’s exact tests for categorical variables with small cell sizes. Statistical significance was determined using α = 0.05.

#### 2.3.3. Qualitative Analysis of Open-Text Question

Respondents had the opportunity to respond to an optional open-text question, “Describe in your own words how your children 12 and under use children’s makeup and body products”. Qualitative analysis of the free response question helped to do the following: (1) learn more about what kind of CMBPs the children of participants use; (2) understand motivations behind CMBP use; (3) explain gaps in quantitative data. Responses were preliminarily explored to develop codes covering all mentioned topics using an inductive approach. A codebook was created to specify codes and their definitions (Table S1). Researchers KK and EM individually assigned each response relevant codes. Multiple codes could be assigned to one response. Once initial coding was complete, researchers compared codes and discussed any disagreements until a consensus was reached. Final codes were applied to responses using NVivo Qualitative Data Analysis Software (Version 12) [27]. Codes were placed hierarchically under broader themes to identify core concepts. *Play*, *Beauty*, and *Practical purpose* codes were grouped underneath *Motivations for use. Health concerns* and *Adult supervision* codes were grouped underneath *Safety*. *Adult influence* and *Marketing* codes were grouped under *Influence and introduction*. *Drug* and *Not makeup/body product* codes were grouped underneath *Other products*. Finally, *No new info* and *Not enough information* codes were grouped underneath *Not usable*. Responses coded under *Not usable* were discarded for analysis.

To understand which words were most commonly used by study participants, a word frequency query was run using NVivo. The word frequency query detailed the ten most common words with a minimum character count of 3 and grouped by exact matches, stemmed words, and synonyms. For example, the word “play” was grouped with “fun, game, games, performances, play, playing, and plays”. As a sensitivity analysis, the word frequency query was conducted with responses coded with *Other products* (referring to products not considered makeup and body products) included as well as excluded.

## 3. Results

### 3.1. Participant Characteristics

**Figure 1:**
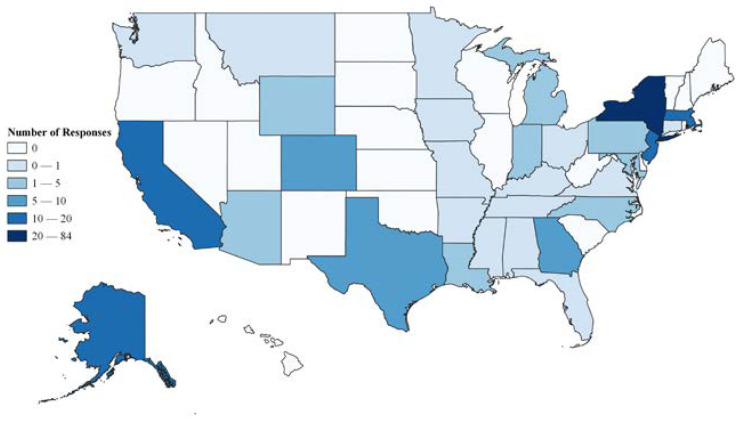
Geographic Distribution of Survey Responses. Categories of response numbers were created using Jenks Optimization.

Survey responses included participants residing in 32 states. The most common state of residence for survey respondents was New York, followed by California, Alaska, Massachusetts, and New Jersey (Figure 1). Characteristics of the parents/guardians who participated in the survey are summarized in Table 1. Of respondents, 54% resided in urban areas, followed by 29% in suburban areas, and 14% in rural communities. Most parents/guardians were younger than 50, with the majority (54%) being 30–39 years old. The majority of respondents identified as female (75%) and White (53%). One-third of the respondents (*n* = 67) identified as Hispanic/Latinx; 61 of these individuals did not select a race category provided. The majority of respondents had at least some college education (62%) but not graduate school, and the distribution of household income varied considerably (from <$25 k per year to $175 k+ per year). Most respondents had one (60%) or two (31%) children aged ≤12 years, and 79% of respondents reported that any of their children aged ≤12 years have used CMBPs.

### 3.2. Child Characteristics

Child characteristics for 312 children aged ≤12 years reported by 207 parent/guardian respondents are shown in the “Total” column of Table 2. Child age was relatively evenly distributed, with the fewest children being 10–12 years old (18%). Respondents reported 52% of the children identify as female and 47% of children identify as White. Similar to parent/guardian respondents, approximately one-third of the child sample did not identify a race and solely identified with Hispanic/Latinx ethnicity.

To understand how children who use makeup and body products may differ from children who do not, respondents were asked if each child in their household aged ≤12 years has used CMBPs. Responses to this question are shown according to child demographics in Table 2. Overall, 219 out of the 312 children (70%) were reported to have ever used CMBPs. A significantly greater proportion of older children used CMBPs than younger children (*p* = 0.011). Notably, the majority of children aged 0–3 years (57%) were reported to use CMBPs. A significantly greater proportion of female children used CMBPs than male children (*p* = 0.014). A greater proportion of White and non-Hispanic children used CMBPs than Black and Hispanic/Latinx children (*p* = 0.038, *p* = 0.051, respectively). 

### 3.3. Products Used

We then investigated CMBP usage patterns and behaviors in children that use CMBPs. Results are shown in Table 3, with responses missing relevant sociodemographic characteristics excluded (*n* = 2) for a sample of 217 children. Because CMBPs represent a broad swath of items, we wanted to understand what type of CMBPs children are using and asked respondents to identify products according to the part of the body for which they are made (see definitions in Methods). The category “body” for products that can be applied all over was most commonly identified (60% of children). Hair and face products were the next most common categories with 44% and 41% of children reported to use them, respectively. Approximately one-third of children were reported to use nail, fragrance, and lip products. The least frequently used category was eye products (18%). Recognizing that children may also use makeup and body products created for adults, we investigated the proportion of products used by children that are specifically made for and marketed toward children. Of the children that use CMBPs, 36% used mostly children’s products. This indicates that most children using CMBPs are also exposed to makeup and body products created for adult use.

**Figure 2:**
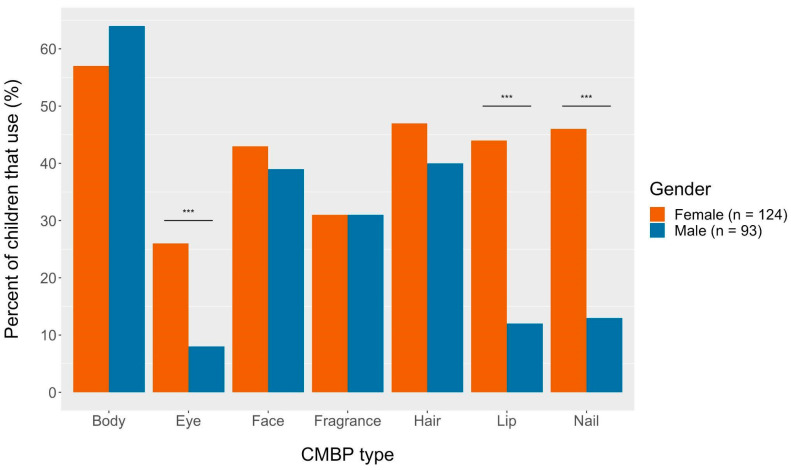
CMBP type by child gender. Chi-square tests conducted for each product by child gender. Female children include cis- and transgender female children (as do male). *** *p*-value < 0.0001.

To investigate possible differences in CMBP use by gender, we investigated the prevalence of use for different types of CMBP for male and female children (Figure 2). Of the children that used CMBPs, male and female children used body, face, fragrance, and hair products at similar rates. A significantly greater proportion of female children used eye, lip, and nail products than male children (*p* < 0.0001).

### 3.4. Child Exposure

To further characterize CMBP exposure, we investigated the frequency and duration of CMBP use, product ingestion, and reading of product ingredients (Table 3). Regarding the frequency of CMBP use, 43% of children rarely used CMBPs (a few times a year or less) while 54% of children used CMBPs at least monthly, and 12% used CMBPs daily, indicating more regular exposure to ingredients. The average duration of CMBP use was also investigated to gain more insight into exposure to CMBP ingredients. Almost half of the children (48%) typically used CMBPs for less than 4 h, and 22% of the children typically used CMBPs for 8 or more hours, indicating lengthier exposures to CMBPs. Also relating to CMBP ingredient exposure, approximately one-third of children (31%) were reported to (unintentionally or intentionally) ingest CMBPs in the last year, suggesting the oral route of exposure to CMBPs is important to consider in addition to dermal. Survey questions querying who applies CMBPs to children may provide further insight into how these products are used. CMBPs were most commonly reported to be applied by a parent or another adult (91%). The child themself or another child was reported to apply CMBPs for 62% of children. Finally, almost half of parents/guardians (46%) reported reading ingredients of CMBPs “often” or “always” before purchasing.

### 3.5. Product Usage Behaviors and Motivations

**Figure 3:**
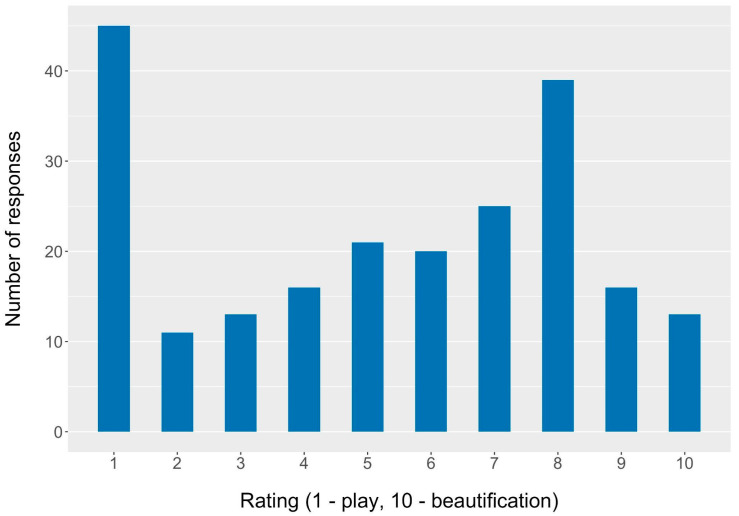
CMBP-use motivation scale (play–beautification rating) histogram (*n* = 219).

The settings and situations in which CMBPs are used provide insights into motivations for CMBP use (Table 3). Just over half (51%) of children were reported to use CMBPs in group or solo play, with group play being more common. Nearly half (45%) of children were reported to use CMBPs in celebrations. Over one-third (37%) of children were reported to wear CMBPs for day-to-day activities. Two-thirds (65%) of children were reported to wear CMBPs outside of their home “never”, “rarely”, or “sometimes”, while 35% of children were reported to wear CMBPs outside of their home “often” or “always”. Respondents were asked to rank on a scale of 1–10 (1 being all play and 10 being all beautification) how their child used CMBPs in the past year. The distribution of responses is shown in Figure 3. The mean response was 5.31; the median was 6.0. The most common response was 1 (21%), indicating that these children use CMBPs for all play purposes. The second most common response was 8 (18%), suggesting these children use CMBPs largely for beautification reasons.

**Figure 4:**
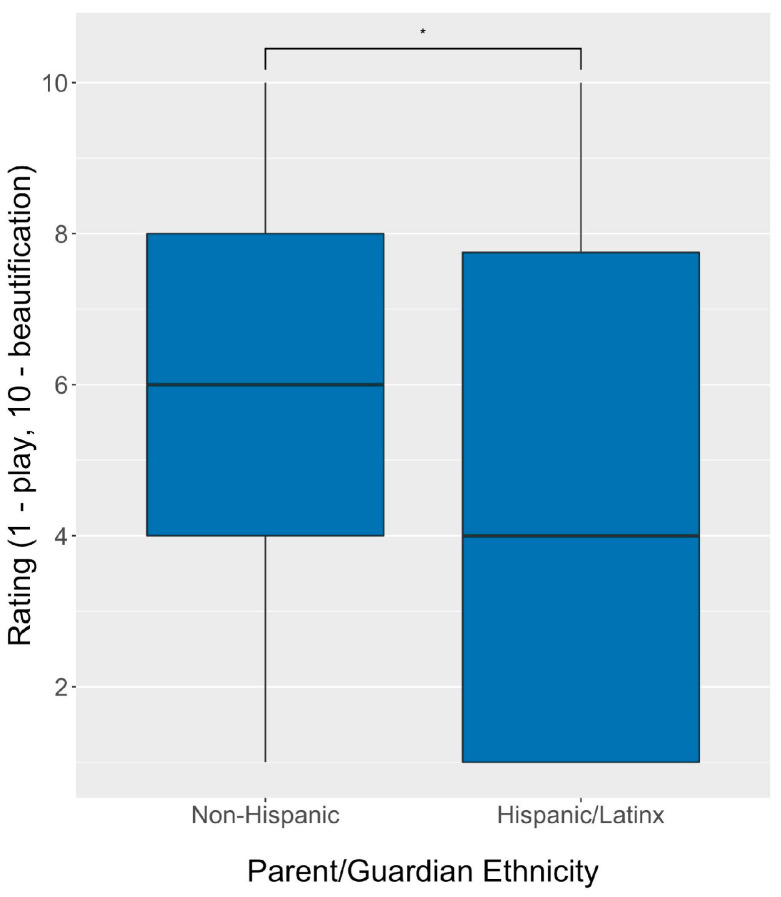
CMBP-use motivation scale (play–beautification rating) by parent/guardian ethnicity (*n* = 217). * *p*-value = 0.039.

Because differences in cosmetic product use by race and ethnicity have been previously reported in the literature, we also investigated whether there were differences in CMBP usage behaviors by parent/guardian ethnicity (Table 3). Significant differences between the types of products used, exposure measures, and motivations for use were found between Hispanic/Latinx respondents and non-Hispanic respondents. A significantly greater proportion of children of Hispanic/Latinx parents/guardians used lip, fragrance, and hair products (*p* < 0.01 for all). Children of Hispanic/Latinx parents/guardians were reported to use CMBPs more frequently (*p* = 0.047) but for shorter durations (*p* < 0.001) than children of non-Hispanic parents/guardians. Hispanic/Latinx parents/guardians reported significantly lower child ingestion rates of CMBPs (*p* < 0.001) and reported reading ingredients of CMBP less frequently before purchasing (*p* = 0.01) than non-Hispanic parents/guardians. For the settings of product use, significantly more children of Hispanic/Latinx parents/guardians used CMBPs in solo play and day-to-day activities, and significantly less used them in performances than children of non-Hispanic/Latinx parents/guardians. Hispanic/Latinx parents/guardians also reported a significantly lower rating on the play–beautification scale, indicating their children used CMBPs more for play purposes (*p* = 0.039, Figure 4) than children of non-Hispanic parents/guardians.

CMBP-use behaviors were also analyzed by child age. For children who used CMBPs, most survey questions did not yield significantly different results by child age (Table S2). The only significant differences found were that a greater proportion of older children used CMBPs applied by themselves and used CMBPs during performances (Table S2). A greater proportion of younger children wore CMBPs applied by a sibling (Table S2).

### 3.6. Product Purchasing Behaviors

Means of child introduction to CMBPs and where parents/guardians report purchasing CMBPs are shown in Table 4. Children were introduced to CMBPs in a wide variety of ways. Introductions through online media (34%) were reported to occur more often than traditional media (23%) and about as often as store displays (37%). Parents/guardians also reported buying CMBPs at a variety of places, most commonly large online retailers (49%). 

### 3.7. Qualitative Analysis of the Open-Text Question

**Figure 5:**
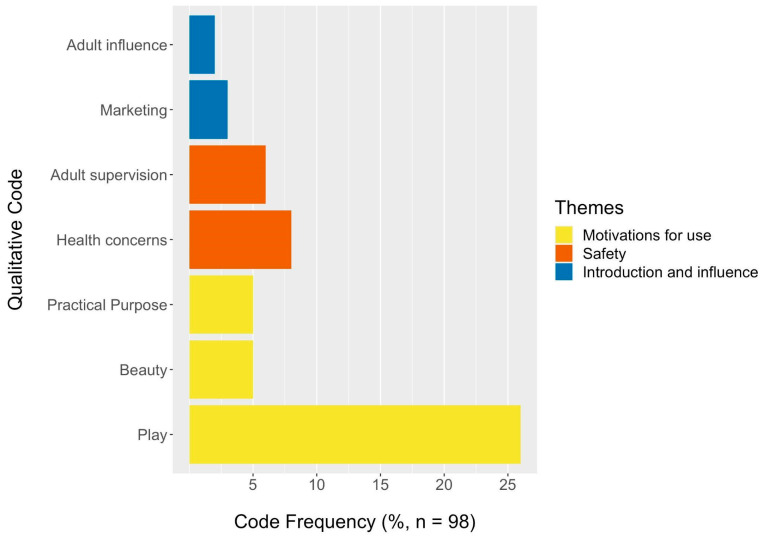
Frequency of thematic codes for responses to “Describe in your own words how your children 12 and under use makeup and body products”.

Approximately half (*n* = 98) of survey participants responded to the optional open-text question, “Describe in your own words how your children 12 and under use makeup and body products”, and responses are described in Figure 5. The demographics of respondents stratified by whether or not they answered the open-text question are shown in Table S3. There was no significant difference in the play–beautification rating between respondents that answered the open-text question and respondents that did not (Table S3).

The most commonly applied code was *Play* which indicates responses that suggest CMBPs are used for fun or during explicitly play activities. One quarter (26%) of responses were coded as *Play* as opposed to alternative motivations for use, *Beauty* and *Practical purpose,* which were both applied to only 5% of responses. Additionally, 7 out of 11 question responses (64%) of the open-text responses provided in Spanish were coded as *Play* in comparison to 22% of the open-text responses provided in English.

The results of the word frequency query conducted in NVivo found “play” and related synonyms to be the third most commonly used word behind “use” and “products”, supporting the qualitative code frequency results. The word frequency query results were consistent when run with and without responses coded with the theme *Other products.* A total of 14% of responses included products that either were not makeup and body products (1%) or products that could reasonably be assumed to be classified as drugs by the FDA and were, therefore, not targeted by this study (13%).

Here are two examples of responses coded as *Play*:

*They mostly use it at home, when they are playing dress up and pretend play. Occasionally, they might get temporary tattoos or chalk hair paint at birthday parties.*

*She loves to play glamour girls!*

Moreover, 14% of parents/guardians commented on CMBP safety in the open-text question. *Health concerns* and *Adult supervision* codes were applied to 8% and 6% of responses, respectively. This is an example of a response coded as *Health concerns:*

*I do not believe in children wearing make up at a young age, even if it is playing, due to the possible dangerous chemicals. As such, the only type of makeup and body products permitted to be used by my children are nail polish and temporary tattoos.*

A small proportion of responses provided information about what influences the children’s use of CMBPs. Two responses were coded with *Adult influence* because they commented on parent/guardian usage of adult makeup and body products influencing the child’s use of CMBPs, and three were coded with *Marketing* because they commented on their children’s attraction to the way CMBPs are sold. Here is an example of a response coded as *Marketing:*

*They also LOVE temporary tattoos and especially love to buy them from the vending machines at the grocery store. The marketing/packaging of those machines is super attractive to them.*

## 4. Discussion

With increasing evidence of harmful ingredients often included in adult cosmetics and CMBPs and children’s biological susceptibility to the effects of toxicants, it is important to uncover how makeup and body products are being used by children to characterize risk and improve safety. This study investigated the use of CMBPs among children aged ≤12 years in the United States through a parent/guardian survey. This study integrated quantitative and qualitative survey responses to assemble rich information regarding children’s use of CMBPs. We found that the majority of children used CMBPs and are thus exposed to their ingredients. Older, female, White, and non-Hispanic children used CMBPs at higher rates than younger, male, Black, and Hispanic/Latinx children. Differences in CMBP-use behaviors were reported by child gender and parent/guardian ethnicity. A lower proportion of male children used eye, lip, and nail products than female children. Children of Hispanic/Latinx parents/guardians used CMBPs more often but for shorter durations and were more likely to use lip, hair, and fragrance products than children of non-Hispanic parents/guardians. About half of all the children were reported to use CMBPs for play, and children of Hispanic/Latinx parents/guardians reported more play motivations for CMBP use than children of non-Hispanic parents/guardians.

### 4.1. Products Used

While makeup and body products are typically associated with adult use, 70% of children included in our survey used CMBPs. A greater proportion of older children used CMBPs than younger children. A majority of children using CMBPs justifies further investigation into how these products are being used. Body products such as face paint were the most commonly used (60%), but a considerable proportion of children also used hair (44%), face (41%), nail (32%), lip (30%), fragrance (30%), and eye products (18%). In comparison, a previous study of children aged ≤14 years in Brazil reported that a greater proportion used perfume (66%) than the proportion of our sample that used fragrances; a similar proportion used hair gel (36%) compared to hair products in our sample; and a smaller proportion used nail polish (22%) than nail products in our sample. Differences between the findings may be explained by country-level differences, the age of the study population, sampling variation, and different product categories/definitions [28].

A significantly greater proportion of children of Hispanic/Latinx parents/guardians used lip, fragrance, and hair products compared to children of non-Hispanic parents/guardians. Fragrances, in particular, may contain ingredients such as phthalates which can exert toxicity yet are not disclosed on their labels [29]. We found that overall female children were more likely to use CMBPs than male children, but that CMBP use varied by gender depending on the product type. Male and female children used body, face, fragrance, and hair products at similar frequencies. Significantly more female children used eye, lip, and nail products compared to male children. These results emphasize that while female children experience a disproportionate exposure to makeup and body product ingredients; safety for children of all genders regarding CMBP use is relevant.

We found that a greater proportion of White children used CMBPs than Black children; however, the previous literature has reported greater usage of cosmetics among women and children of color [18,21]. It is possible that the patterns of cosmetic use by race are different for children’s products than adult products. Additionally, the sample size of Black children in this study was small and may not be representative. It is also important to note that we did not include products such as hair relaxers and skin-lightening creams in our definition of CMBPs, which are known to contribute to this disparity [18,19,20].

### 4.2. Child Exposure

In light of the limited regulation of cosmetics and evidence of toxicity associated with common ingredients, we investigated the frequency and duration of CMBP use. More than half of the children of respondents used CMBPs at least monthly and approximately one-fifth typically use CMBPs for 8 h or more. Frequent and lengthy exposures to CMBPs in early life warrant concern about the safety of their ingredients. Child behaviors such as hand-to-mouth activity and inappropriate application could further increase exposure to harmful ingredients. One-third of children were reported to have ingested CMBPs in the last year, and many children were reported to wear CMBPs applied by another child, increasing the risk of incorrect application and exposure to harmful ingredients. Our results suggest that the risk of exposure to CMBP ingredients may be affected by ethnicity, as children of Hispanic/Latinx parents/guardians were reported to use CMBPs significantly more frequently but for shorter durations and to ingest CMBP at lower rates than children of non-Hispanic parents/guardians. A limitation of investigating the average length of CMBP use across all products is that some products (e.g., nail polish) are intended to be used on longer time scales (i.e., weeks as opposed to hours).

Almost half of the respondents reported reading the ingredients of CMBPs “often” or “always” before purchasing them, suggesting that parents/guardians are interested in the composition and safety of makeup and body products they buy for their children. Some responses to the open-text question reflected this concern. These results are particularly interesting when considering that large online retailers were the most commonly reported point of purchase for CMBPs. According to the Fair Packaging and Labeling Act, ingredient lists are required to be listed on physical product packaging but not necessarily posted on websites where cosmetics are sold online [3]. This represents a major gap in regulation for a prevalent and likely growing purchasing channel for adult cosmetics and CMBPs. Lockdowns and risks associated with crowded public places during the ongoing COVID-19 pandemic may have increased the purchasing of CMBPs online. While parents/guardians may report reading the ingredients, we did not assess whether or how they screen the safety of ingredients. It is also possible that the high proportion of respondents who reported reading ingredient lists was influenced by social desirability bias in light of prior knowledge about potentially harmful ingredients in cosmetics, believing that it is more socially acceptable to read ingredients before purchasing. Regardless, the authors believe the responsibility for CMBP safety should be on regulatory bodies as opposed to consumers. 

### 4.3. Product Usage Behaviors and Motivations

Children may use CMBPs for different reasons than adults, who primarily use makeup and body products for beautification. However, CMBPs lack rigorous regulation and may contain similar toxic ingredients as adult products. It was hypothesized that children use CMBPs primarily for play as opposed to beautification. Slightly more than half of the children of respondents were reported to use CMBPs in group or solo play settings. When parents/guardians were asked specifically to rank their children’s motivation behind the use of CMBP from 1 (play) to 10 (beautification), the median result was 6. Interestingly, the most frequent response was 1, suggesting that a subset of children of the respondents use CMBPs exclusively for play. Corroborating these findings, the majority of children were reported to wear CMBPs outside of their home “never”, “rarely”, or “sometimes”, indicating a higher likelihood that CMBPs were being used for play. In the qualitative analysis of the open-text question, *Play* was the most commonly applied code, and “play” and its synonyms were the most commonly used substantive words. Less than half of the respondents chose to fill out the optional open-text question, so response bias may be present. However, there was no significant difference in the play–beautification rating between the response groups. Overall, the quantitative and qualitative responses both indicate that a substantial proportion of children use CMBPs for play.

Our results also suggest that the motivations for the use of CMBPs may vary by ethnicity. More frequent but shorter uses of CMBPs were reported for children of Hispanic/Latinx parents/guardians and may be indicative of CMBPs being used in play times. The play–beautification rating supports this finding as children of Hispanic/Latinx parents/guardians had a significantly lower average rating than children of non-Hispanic parents/guardians. The qualitative results further support the idea that children of Hispanic/Latinx parents/guardians use CMBPs for play with a large majority of Spanish responses coded as *Play* as compared to a minority of English responses—though the number of respondents that took the survey in Spanish was low.

While understanding motivation for use is important for providing additional context for regulation, the use of CMBPs for any purpose still results in exposure to potentially harmful ingredients. Furthermore, we found that the majority of children use more adult makeup and body products than children’s products. The investigation of adult cosmetic products was outside the scope of this study, but it is important to be aware of children’s exposure to products created for adult use.

### 4.4. Strengths and Limitations

While there is a growing body of literature about cosmetic product exposure, few studies have investigated children’s products specifically. This survey asked parents/guardians about their children’s experiences using makeup and body products created for children, providing information about early life exposures to this unique class of products. This study utilized mixed methodologies, combining quantitative analysis of multiple-choice questions with a qualitative exploration and thematic analysis of the open-text question. This enabled the examination of measurable product-use behaviors within the context of parent/guardian perspectives in their own words.

Broad eligibility criteria for study participation and the availability of the survey in multiple languages allowed us to obtain information about CMBP use potentially relevant to many different localities and communities, despite having a relatively small sample size. Although we received responses from across the United States, the study sample was not nationally representative. For example, one of the states with the highest number of responses was Alaska. We expect oversampling of particular social networks to be a result of chain referrals and online survey distribution. Because the topic of the study was shared on recruitment materials, it is also possible parents/guardians with children that do not use CMBPs may be underrepresented. The relatively small sample size for some groups also led to decreased statistical power for detecting differences in CMBP use across demographics. For example, the analysis of CMBP-type usage by child gender did not include non-binary children so as not to make conclusions based on extremely small sample sizes. Respondent race groups were also small, so sub-analyses were restricted to ethnicity. Furthermore, because the analyses were mainly descriptive, clustering within families (i.e., siblings) was not accounted for. Qualitative analysis by survey language was also limited due to the small number of responses received in Spanish (*n* = 11). Additionally, this study aims to understand the context in which children use CMBPs, but parents/guardians completed the survey, and they may not fully understand why and how their children use such products, especially outside of the home. Because the survey was administered through a personal device, parents/guardians without a device with internet access are also not represented.

A small proportion (14%) of the open-text question responses referred to products that were not included in the definition of CMBPs provided for this study, suggesting that some respondents misunderstood relevant products when completing the survey. However, the word frequency query conducted for responses to the open-text question did not show any differences when such products were included in the analysis or not, suggesting the results of at least the qualitative analysis were not strongly influenced by these additional products—mostly makeup and body products that also have medicinal purposes and, thus, can be characterized as a drug. A limitation of the word frequency query is the synonyms that the Nvivo software (Version 12) automatically uses. For example, “performances” was considered a synonym for “play” although we consider CMBP use for performances (e.g., dance) to be distinct from play. On the other hand, “pretend” was not attributed as a synonym for “play” even though it could be considered one representing imaginative play. Promisingly, *Play* was also the most commonly applied code when the context of the entire response was taken into account by researchers.

## 5. Conclusions

This mixed-method study investigated the use of CMBPs among children ≤12 years of age. We explored what types of products are used and the frequency, duration, and motivations for their use as well as gathered information about where and how these products are purchased and whether parents/guardians typically look at ingredient lists. Overall, we found that the majority of children use CMBPs and that a substantial proportion of children use CMBPs for play. These findings suggest that children’s motivations for the use of CMBPs may differ from the beautification motivations that underlie the use of adult cosmetics. Some differences in CMBP use by child gender and parent/guardian ethnicity were found. Given the few existing studies about children’s exposure to CMBPs, this relatively small survey study is a good preface for future, larger-scale studies which could further address children’s use of both adult products and children’s products, as well as concerns regarding toxicity and environmental injustice.

## Figures and Tables

**Table 1:** Parent/guardian characteristics.

	Overall (*n* = 207)
Survey Language	
English	181 (87%)
Spanish	26 (13%)
Type of Community	
Urban	112 (54%)
Suburban	59 (29%)
Rural	29 (14%)
Parent/Guardian Age	
<30	41 (20%)
30–39	112 (54%)
40–49	45 (22%)
50+	8 (4%)
Parent/Guardian Gender	
Female	155 (75%)
Cisgender Female	154 (74%)
Transgender Female	1 (0.5%)
Male	49 (24%)
Cisgender Male	49 (24%)
Transgender Male	0 (0%)
Two-spirit	1 (0.5%)
Gender Non-binary	0 (0%)
Parent/Guardian Race	
White	110 (53%)
Black/African American	14 (7%)
Asian	9 (4%)
South Asian	5 (2%)
East Asian	4 (2%)
American Indian/Alaska Native	8 (4%)
Native Hawaiian/Pacific Islander	1 (0.5%)
Middle Eastern/North African	0 (0%)
2 or more races	2 (1%)
American Indian/Alaska Native, White	1 (0.5%)
American Indian/Alaska Native, East Asian	1 (0.5%)
Parent/Guardian Ethnicity	
Non-Hispanic	138 (67%)
Hispanic/Latinx	67 (32%)
Parent/Guardian Education	
High School or less	27 (13%)
Some College/College	128 (62%)
Some Graduate School or more	51 (25%)
Household Income	
Less than $25 k	22 (11%)
$25 k–$75 k	58 (28%)
$75 k–$125 k	60 (29%)
$125 k–$175 k	24 (12%)
$175 k+	30 (15%)
Number of Children 12 and under	
1	125 (60%)
2	64 (31%)
3	14 (7%)
4	3 (1%)
5	1 (0.5%)
Have any of your children ages 12 and younger ever used children’s makeup and body products?	
No	44 (21%)
Yes	163 (79%)

Missing data (selected “Prefer not to answer”): Type of Community, *n* = 7; Parent/Guardian Age, *n* = 1; Parent/Guardian Gender, *n* = 2; Parent/Guardian Race, *n* = 63; Parent/Guardian Ethnicity, *n* = 2; Parent/Guardian Education, *n* = 1; Household Income, *n* = 13.

**Table 2:** Child characteristics by CMBP use.

Has This Child Ever Used Children’s Makeup and Body Products?	No (*n* = 93) *n* (Row %)	Yes (*n* = 219) *n* (Row %)	Total (*n*= 312) *n* (Col %)	*p*-Value
Child Age				
0–3	36 (43%)	48 (57%)	84 (27%)	0.011 *
4–6	23 (26%)	64 (74%)	87 (28%)	
7–9	17 (20%)	68 (80%)	85 (27%)	
10–12	17 (20%)	39 (80%)	56 (18%)	
Child Gender				
Female	39 (24%)	124 (76%)	163 (52%)	0.014 *^,†^
Cisgender Female	39 (24%)	124 (76%)	163 (52%)	
Transgender Female	0 (0%)	0 (0%)	0 (0%)	
Male	54 (37%)	93 (63%)	147 (47%)	
Cisgender Male	54 (38%)	90 (63%)	144 (46%)	
Transgender Male	0 (0%)	3 (100%)	3 (1%)	
Non-binary	0 (0%)	1 (100%)	1 (0.5%)	
Child Race				
White	32 (22%)	113 (78%)	145 (47%)	0.038 *^,‡^
Black/African American	14 (39%)	22 (61%)	36 (12%)	
Asian	3 (30%)	7 (70%)	10 (3%)	
South Asian	1 (17%)	5 (83%)	6 (2%)	
East Asian	2 (50%)	2 (50%)	4 (1%)	
American Indian/Alaska Native	1 (11%)	8 (89%)	9 (3%)	
Native Hawaiian/Pacific Islander	0 (0%)	1 (100%)	1 (1%)	
Middle Eastern/North African	0 (0%)	0 (0%)	0 (0%)	
2 or more races ^a^	2 (20%)	8 (80%)	10 (3%)	
Child Ethnicity				
Non-Hispanic	46 (24%)	145 (76%)	191 (61%)	0.051
Hispanic/Latinx	38 (35%)	72 (65%)	110 (35%)	

Missing data (selected “Prefer not to answer”): Child Gender, *n* = 1; Child Race, *n* = 101; Child Ethnicity, *n* = 11. *p*-values were generated from Chi-square tests. † Chi-square compared male and female children. ‡ Chi-square compared Black and White children. ^a^ While 0% of children were reported to be solely Middle Eastern/North African, some children that were reported to identify with 2+ races selected one as Middle Eastern/North African. * *p*-value < 0.05.

**Table 3:** CMBP use by parent ethnicity.

	Non-Hispanic (*n* = 143) *n* (Col %)	Hispanic/Latinx (*n* = 74) *n* (Col %)	Total (*n* = 217) *n* (Col %)	*p*-Value
Type(s) of CMBP used ^a^				
Body	90 (63%)	40 (54%)	130 (60%)	0.243
Hair	53 (37%)	42 (57%)	95 (44%)	0.006 *
Face	64 (45%)	25 (34%)	89 (41%)	0.146
Nail	48 (34%)	21 (28%)	69 (32%)	0.539
Lip	34 (24%)	31 (42%)	65 (30%)	0.008 *
Fragrance	31 (22%)	35 (47%)	66 (30%)	<0.001 *
Eye	25 (18%)	14 (19%)	39 (18%)	0.853
None	3 (2%)	1 (1%)	4 (2%)	1
Proportion of makeup and body products used that are child products (vs. adult products)				
None (0%)	4 (3%)	2 (3%)	6 (3%)	0.267
Few (Less than 25%)	49 (34%)	24 (32%)	73 (34%)	
Less than Half (25–50%)	40 (28%)	12 (16%)	52 (24%)	
More than Half (50–75%)	23 (16%)	10 (14%)	33 (15%)	
Large Majority (75–100%)	25 (18%)	20 (27%)	45 (21%)	
Frequency of CMBP Use				
Once a year or less	13 (9%)	7 (10%)	20 (9%)	0.047 *
A few times a year	52 (36%)	22 (30%)	74 (34%)	
Monthly	32 (22%)	9 (12%)	41 (19%)	
Once every two weeks	15 (11%)	5 (7%)	20 (9%)	
Weekly	20 (14%)	11 (15%)	31 (14%)	
Daily or more	11 (8%)	16 (22%)	27 (12%)	
Duration of CMBP Use				
0–2 h	21 (15%)	33 (45%)	54 (25%)	<0.001 *
2–4 h	38 (27%)	11 (15%)	49 (23%)	
4–6 h	30 (21%)	7 (10%)	37 (17%)	
6–8 h	15 (11%)	2 (3%)	17 (8%)	
8+ h	30 (21%)	17 (23%)	47 (22%)	
Ingestion of CMBP				
Yes	59 (41%)	8 (11%)	67 (31%)	<0.001 *
No	73 (51%)	61 (82%)	134 (62%)	
Who applies CMBP ^a^				
Child	92 (64%)	43 (58%)	135 (62%)	
Child	51 (36%)	26 (35%)	77 (36%)	1
Sibling	22 (15%)	11 (15%)	33 (15%)	1
Friend	19 (13%)	6 (8%)	25 (12%)	0.37
Adult	128 (90%)	69 (93%)	197 (91%)	
Parent	83 (58%)	51 (69%)	134 (62%)	0.141
Caregiver	18 (13%)	4 (5%)	22 (10%)	0.153
Event	27 (19%)	14 (19%)	41 (19%)	1
Frequency respondent reads CMBP ingredients before purchasing				
Never	17 (12%)	6 (8%)	23 (11%)	0.01 *
Rarely	15 (11%)	17 (23%)	32 (15%)	
Sometimes	29 (20%)	18 (24%)	47 (22%)	
Often	51 (36%)	12 (16%)	63 (29%)	
Always	22 (15%)	14 (19%)	36 (17%)	
Frequency of CMBP wear outside the home				
Never	2 (1%)	7 (10%)	9 (4%)	0.055
Rarely	33 (23%)	18 (24%)	51 (24%)	
Sometimes	54 (38%)	26 (35%)	80 (37%)	
Often	34 (24%)	11 (15%)	45 (21%)	
Always	19 (13%)	11 (15%)	30 (14%)	
Setting(s) CMBP used in ^a^				
Celebrations	66 (46%)	32 (43%)	98 (45%)	0.774
Day-to-day	44 (31%)	37 (50%)	81 (37%)	0.008 *
Group play	51 (36%)	19 (26%)	70 (32%)	0.168
Solo play	22 (15%)	20 (27%)	42 (19%)	0.047 *
Performances	46 (32%)	7 (10%)	53 (24%)	<0.001 *
Religious events	14 (10%)	3 (4%)	17 (8%)	0.185
Play → Beautification Rating				
Mean (SD)	5.63 (2.73)	4.70 (3.30)	5.31 (2.96)	0.039 *
Median [Min, Max]	6 [1,10]	4 [1,10]	6 [1,10]	

Missing data (did not answer or selected “Do not know”/“Prefer not to answer”): Proportion of makeup and body products used that are child products, *n* = 8; Frequency of CMBP use, *n* = 4; Duration of CMBP use *n* = 13; Ingestion of CMBP, *n* = 16; Who applies CMBP, *n* = 1; Frequency respondent reads CMBP ingredients before purchasing, *n* = 16; Frequency of CMBP wear outside the home, *n* = 2; Settings CMBP used in, *n* = 6. Fisher’s exact test was used for categorical variables, and Wilcoxon test was used for numerical variables (play–beautification rating). ^a^ Categories with percentages that sum to >100% were “check all that apply” questions. * *p*-value < 0.05.

**Table 4:** Introduction and Purchase Locations.

	(*n* = 163) *n* (Col %)
Means of child introduction to CMBP ^a^	
Store display	60 (37%)
Another person	59 (36%)
Online media	55 (34%)
Activity (e.g., dance class, theater)	42 (26%)
Celebrations	35 (22%)
Traditional media	37 (23%)
Unknown	5 (3%)
Purchase Locations ^a^	
Large online retailer (e.g., Amazon)	79 (49%)
Small online retailer (e.g., a boutique)	44 (27%)
Big box retailer (e.g., Walmart/Target)	60 (37%)
Pharmacy (e.g., CVS/Walgreens)	51 (31%)
Children’s retailer (e.g., Claire’s, Justice)	33 (20%)
Dollar store	9 (6%)

^a^ Categories with percentages that sum to >100% were “check all that apply” questions.

## Data Availability

The data presented in this study are available on request from the corresponding author. The data are not publicly available due to ethical restrictions.

## Supplementary Materials

The following supporting information can be downloaded at: https://www.mdpi.com/article/10.3390/ijerph20032114/s1: Table S1. Qualitative analysis of open-text question codebook; Table S2. CMBP-use behaviors by child age. Fisher’s exact test was used for categorical variables and Kruskal–Wallis test was used for numerical variables (play–beautification rating); Table S3. Parent/guardian demographics by response to open-text question. Fisher’s exact test was used for categorical variables and Wilcoxon test was used for numerical variables (play–beautification rating).
