# Supplementary material for: Usage of Children’s Makeup and Body Products in the United States and Implications for Childhood Environmental Exposures
Source: Int J Environ Res Public Health. 2023 Jan 24;20(3):2114. doi: 10.3390/ijerph20032114 (PMC9915933; doi:10.3390/ijerph20032114)
Supplement: Supplementary file 1 [file ijerph-20-02114-s001.zip › ijerph-2161775-supplementary.pdf]

International Journal of
Environmental Research and Public Health

MDPI

Supplementary Materials

Usage of Children's Makeup and Body Products in the United States and Implications for Childhood Environmental Exposures

Eleanor A. Medley [1,†], Kendall E. Kruchten [1,†], Miranda J. Spratlen [1], Maricela Ureño [1], Anabel Cole [1], Rashmi Joglekar [2] and Julie B. Herbstman [1,*]

[1] Columbia Center for Children's Environmental Health, Department of Environmental Health Sciences, Columbia University Mailman School of Public Health, New York, NY 10032, USA
[2] Earthjustice, Toxic Exposure and Health Program, Washington, DC 20001, USA
* Correspondence: jh2678@cumc.columbia.edu
† These authors contributed equally to this wrok.

Table S1. Qualitative analysis of open text question codebook.

Theme	Code Name	When to Apply
Motivations for use	Play	Response suggests child uses children's makeup and body products for fun or during explicitly play activities
	Beauty	Response suggests child uses children's makeup and body products for beautification purposes
	Practical Purpose	Response describes practical purpose for using children's makeup and body products distinct from play or beautification motivations (e.g. using nail polish to stop nail biting habit, using hair detangler for large knots)
Safety	Health concerns	Response expresses concern about the health implications of children's makeup and body products
	Adult supervision	Response mentions adult supervision of product use
Introduction and influence	Adult influence	Response describes how adult use of cosmetic products affects child's use of children's makeup and body products
	Marketing	Response mentions influence of marketing/packaging of children's makeup and body products on their child
Other products	Drug	Response identifies a product that can reasonably be assumed to be categorized as a drug based on the FDA definition (Not applicable to this investigation)

	Not makeup/body product	Response describes a product that is not makeup nor a body product
Not usable	No new info	Response reiterates response to previously asked multiple choice question and does not provide any additional information
	Not enough information	Not enough information present in the response to code

Table S2. CMBP use behaviors by child age. Fisher's exact test was used for categorical variables and Kruskal-Wallis test was used for numerical variables (play-beautification rating).

	0-3 (*n*=48)	4-6 (*n*=64)	7-9 (*n*=68)	10-12 (*n*=39)	Total (*n*=219)	*p*-Value
**Type of CMBP used**						
Body	26 (54.2%)	32 (50.0%)	49 (72.1%)	23 (59.0%)	130 (59.4%)	0.057
Hair	19 (39.6%)	26 (40.6%)	34 (50.0%)	17 (43.6%)	96 (43.8%)	0.648
Face	13 (27.1%)	26 (40.6%)	32 (47.1%)	18 (46.2%)	89 (40.6%)	0.145
Nail	10 (20.8%)	26 (40.6%)	22 (32.4%)	12 (30.8%)	70 (32.0%)	0.174
Lip	14 (29.2%)	15 (23.4%)	25 (36.8%)	12 (30.8%)	66 (30.1%)	0.424
Fragrance	15 (31.3%)	18 (28.1%)	23 (33.8%)	11 (28.2%)	67 (30.6%)	0.897
Eye	9 (18.8%)	7 (10.9%)	14 (20.6%)	9 (23.1%)	39 (17.8%)	0.336
None	2 (4.17%)	0 (0%)	1 (1.47%)	1 (2.56%)	4 (1.83%)	0.392
Do not know	1 (2.08%)	0 (0%)	0 (0%)	0 (0%)	1 (0.457%)	0.397
**Proportion of makeup and body products used that are child products (vs. adult products)**						
None (0%)	1 (2.08%)	1 (1.56%)	3 (4.41%)	1 (2.56%)	6 (2.74%)	0.912
Few (Less than 25%)	17 (35.4%)	18 (28.1%)	22 (32.4%)	17 (43.6%)	74 (33.8%)	
Less than Half (25-50%)	10 (20.8%)	18 (28.1%)	16 (23.5%)	8 (20.5%)	52 (23.7%)	
More than Half (50-75%)	9 (18.8%)	11 (17.2%)	8 (11.8%)	6 (15.4%)	34 (15.5%)	
Large Majority (75-100%)	10 (20.8%)	15 (23.4%)	14 (20.6%)	6 (15.4%)	45 (20.5%)	
Do not know	1 (2.08%)	1 (1.56%)	5 (7.35%)	1 (2.56%)	8 (3.65%)	
**Frequency of CMBP Use**						
Once a year or less	8 (16.7%)	6 (9.38%)	6 (8.82%)	1 (2.56%)	21 (9.59%)	0.586
A few times a year	15 (31.3%)	22 (34.4%)	23 (33.8%)	15 (38.5%)	75 (34.2%)	
Monthly	10 (20.8%)	11 (17.2%)	13 (19.1%)	7 (17.9%)	41 (18.7%)	
Once every two weeks	4 (8.33%)	5 (7.81%)	6 (8.82%)	5 (12.8%)	20 (9.13%)	
Weekly	4 (8.33%)	6 (9.38%)	13 (19.1%)	8 (20.5%)	31 (14.2%)	
Daily or more	7 (14.6%)	11 (17.2%)	6 (8.82%)	3 (7.69%)	27 (12.3%)	
Do not know	0 (0%)	3 (4.69%)	1 (1.47%)	0 (0%)	4 (1.83%)	

**Duration of CMBP Use**						
0-2 hours	15 (31.3%)	18 (28.1%)	15 (22.1%)	7 (17.9%)	55 (25.1%)	0.4
2-4 hours	11 (22.9%)	13 (20.3%)	15 (22.1%)	11 (28.2%)	50 (22.8%)	
4-6 hours	10 (20.8%)	8 (12.5%)	11 (16.2%)	8 (20.5%)	37 (16.9%)	
6-8 hours	1 (2.08%)	4 (6.25%)	5 (7.35%)	7 (17.9%)	17 (7.76%)	
8+ hours	10 (20.8%)	15 (23.4%)	17 (25.0%)	5 (12.8%)	47 (21.5%)	
Do not know	1 (2.08%)	6 (9.38%)	5 (7.35%)	1 (2.56%)	13 (5.94%)	
**Ingestion of CMBP**						
Yes	17 (35.4%)	20 (31.3%)	21 (30.9%)	9 (23.1%)	67 (30.6%)	0.888
No	29 (60.4%)	39 (60.9%)	41 (60.3%)	27 (69.2%)	136 (62.1%)	
Do not know	2 (4.17%)	5 (7.81%)	6 (8.82%)	3 (7.69%)	16 (7.31%)	
**Who applies CMBP**						
Child	19 (39.6%)	32 (50.0%)	53 (77.9%)	31 (79.5%)	135 (62%)	
The child themself	4 (8.33%)	12 (18.8%)	38 (55.9%)	23 (59.0%)	77 (35.2%)	<0.001*
Sibling	10 (20.8%)	14 (21.9%)	7 (10.3%)	2 (5.13%)	33 (15.1%)	0.047*
Friend	5 (10.4%)	6 (9.38%)	8 (11.8%)	6 (15.4%)	25 (11.4%)	0.827
Adult	42 (87.5%)	62 (96.9%)	62 (91.2%)	32 (82.1%)	198 (90.4%)	
Parent	29 (60.4%)	44 (68.8%)	40 (58.8%)	22 (56.4%)	135 (61.6%)	0.556
Caregiver	8 (16.7%)	5 (7.81%)	8 (11.8%)	1 (2.56%)	22 (10.0%)	0.145
Event person	5 (10.4%)	13 (20.3%)	14 (20.6%)	9 (23.1%)	41 (18.7%)	0.374
**Frequency with which respondent reads CMBP ingredients before purchasing**						
Never	4 (9.09%)	7 (11.7%)	10 (15.9%)	2 (5.56%)	23 (11.3%)	0.494
Rarely	6 (13.6%)	9 (15.0%)	12 (19.0%)	5 (13.9%)	32 (15.8%)	
Sometimes	7 (15.9%)	15 (25.0%)	19 (30.2%)	7 (19.4%)	48 (23.6%)	
Often	17 (38.6%)	19 (31.7%)	15 (23.8%)	12 (33.3%)	63 (31.0%)	
Always	9 (20.5%)	10 (16.7%)	7 (11.1%)	10 (27.8%)	36 (17.7%)	
Prefer not to answer	1 (2.27%)	0 (0%)	0 (0%)	0 (0%)	1 (0.493%)	
**Frequency of CMBP wear outside the home**						
Never	5 (10.4%)	3 (4.69%)	2 (2.94%)	0 (0%)	10 (4.57%)	0.273

Rarely	7 (14.6%)	20 (31.3%)	16 (23.5%)	9 (23.1%)	52 (23.7%)	
Sometimes	16 (33.3%)	18 (28.1%)	30 (44.1%)	16 (41.0%)	80 (36.5%)	
Often	11 (22.9%)	15 (23.4%)	11 (16.2%)	8 (20.5%)	45 (20.5%)	
Always	9 (18.8%)	6 (9.38%)	9 (13.2%)	6 (15.4%)	30 (13.7%)	
Do not know	0 (0%)	2 (3.13%)	0 (0%)	0 (0%)	2 (0.913%)	
**Settings CMBP used in**						
Celebrations	24 (50.0%)	27 (42.2%)	28 (41.2%)	20 (51.3%)	99 (45.2%)	0.636
Day-to-day activities	15 (31.3%)	21 (32.8%)	27 (39.7%)	18 (46.2%)	81 (37.0%)	0.435
Group Play	14 (29.2%)	22 (34.4%)	20 (29.4%)	14 (35.9%)	70 (32.0%)	0.853
Solo Play	9 (18.8%)	12 (18.8%)	15 (22.1%)	6 (15.4%)	42 (19.2%)	0.879
Performances	4 (8.33%)	17 (26.6%)	18 (26.5%)	14 (35.9%)	53 (24.2%)	0.012*
Religious Events	4 (8.33%)	3 (4.69%)	6 (8.82%)	4 (10.3%)	17 (7.76%)	0.713
Unknown	1 (2.08%)	3 (4.69%)	1 (1.47%)	1 (2.56%)	6 (2.74%)	0.811
**Play → Beautification Rating**						
Mean (SD)	5.67 (3.24)	5.23 (3.22)	4.66 (2.69)	5.95 (2.50)	5.28 (2.97)	0.1042
Median [Min, Max]	6.50 [1.00, 10.0]	6.00 [1.00, 10.0]	5.00 [1.00, 10.0]	7.00 [1.00, 10.0]	6.00 [1.00, 10.0]	

* *p*-value < 0.05.

Table S3. Parent/guardian demographics by response to open text question. Fisher's exact test was used for categorical variables and Wilcoxon test was used for numerical variables (play-beautification rating).

	No text response (*n*=65)	Text Response (*n*=98)	Total (*n*=163)	*p*-Value
**Survey Language**				
English	60 (92.3%)	87 (88.8%)	147 (90.2%)	0.594
Spanish	5 (7.69%)	11 (11.2%)	16 (9.82%)	
**Type of Community**				
Urban	47 (72.3%)	45 (45.9%)	92 (56.4%)	0.005*
Suburban	11 (16.9%)	35 (35.7%)	46 (28.2%)	
Rural	7 (10.8%)	15 (15.3%)	22 (13.5%)	
Prefer not to answer	0 (0%)	3 (3.06%)	3 (1.84%)	
**Parent/Guardian Age**				
<30	18 (27.7%)	13 (13.3%)	31 (19.0%)	<0.001*
30-39	41 (63.1%)	48 (49.0%)	89 (54.6%)	
40-49	5 (7.69%)	32 (32.7%)	37 (22.7%)	
50+	1 (1.54%)	4 (4.08%)	5 (3.07%)	
Prefer not to answer	0 (0%)	1 (1.02%)	1 (0.613%)	
**Parent/Guardian Gender**				
Cisgender Female	44 (67.7%)	80 (81.6%)	124 (76.1%)	0.034*
Transgender Female	0 (0%)	0 (0%)	0 (0%)	
Cisgender Male	21 (32.3%)	16 (16.3%)	37 (22.7%)	
Transgender Male	0 (0%)	0 (0%)	0 (0%)	
Non-binary	0 (0%)	0 (0%)	0 (0%)	
Two Spirit	0 (0%)	1 (1.02%)	1 (0.613%)	
Prefer not to answer	0 (0%)	1 (1.02%)	1 (0.613%)	
**Parent/Guardian Race**				
White	45 (69.2%)	47 (48.0%)	92 (56.4%)	0.11
Black/African American	4 (6.15%)	10 (10.2%)	14 (8.59%)	
South Asian	2 (3.08%)	1 (1.02%)	3 (1.84%)	
East Asian	1 (1.54%)	1 (1.02%)	2 (1.23%)	
American Indian/Alaska Native	1 (1.54%)	7 (7.14%)	8 (4.91%)	
Native Hawaiian/Pacific Islander	0 (0%)	1 (1.02%)	1 (0.613%)	
Middle Eastern/North African	0 (0%)	0 (0%)	0 (0%)	
American Indian/Alaska Native, White	0 (0%)	1 (1.02%)	1 (0.613%)	

American Indian/Alaska Native, East Asian	0 (0%)	1 (1.02%)	1 (0.613%)	
Prefer not to answer	12 (18.5%)	29 (29.6%)	41 (25.2%)	
**Parent/Guardian Ethnicity**				
Non-Hispanic	52 (80.0%)	64 (65.3%)	116 (71.2%)	0.073
Hispanic/Latinx	13 (20.0%)	32 (32.7%)	45 (27.6%)	
Prefer not to answer	0 (0%)	2 (2.04%)	2 (1.23%)	
**Parent/Guardian Education**				
High School or Less	3 (4.62%)	15 (15.3%)	18 (11.0%)	0.016*
Some College/College	50 (76.9%)	54 (55.1%)	104 (63.8%)	
Some Graduate School or more	12 (18.5%)	28 (28.6%)	40 (24.5%)	
Prefer not to answer	0 (0%)	1 (1.02%)	1 (0.613%)	
**Household Income**				
Less than $25k	4 (6.15%)	7 (7.14%)	11 (6.75%)	0.844
$25k - $75k	15 (23.1%)	29 (29.6%)	44 (27.0%)	
$75k - $125k	26 (40.0%)	29 (29.6%)	55 (33.7%)	
$125k - $175k	8 (12.3%)	13 (13.3%)	21 (12.9%)	
$175k+	9 (13.8%)	14 (14.3%)	23 (14.1%)	
Prefer not to answer	3 (4.62%)	6 (6.12%)	9 (5.52%)	
**Number of Children 12 and Under**				
1	48 (73.8%)	52 (53.1%)	100 (61.3%)	0.009*
2	10 (15.4%)	37 (37.8%)	47 (28.8%)	
3	6 (9.23%)	8 (8.16%)	14 (8.59%)	
4	1 (1.54%)	1 (1.02%)	2 (1.23%)	
5	0 (0%)	0 (0%)	0 (0%)	
**Play → Beautification Rating**				
Mean (SD)	5.46 (2.81)	5.168 (3.06)	5.28 (2.97)	0.528
Median [Min, Max]	6 [1, 10]	5 [1, 10]	6 [1, 10]	

* *p*-value < 0.05.
